# Avascular Necrosis of the Femoral Head Following Sliding Hip Screw Fixation in the Context of Recent Contralateral Vascular Surgery: A Report of a Rare Case

**DOI:** 10.7759/cureus.92498

**Published:** 2025-09-16

**Authors:** Gayle Caruana, Andrew Bernard

**Affiliations:** 1 Surgery, Mater Dei Hospital, Msida, MLT; 2 Orthopaedics and Trauma, Mater Dei Hospital, Msida, MLT

**Keywords:** avascular necrosis (avn), femoro-popliteal bypass, painful hip, sliding hip screw, vascular surgery

## Abstract

Avascular necrosis (AVN) of the femoral head is a rare complication following extracapsular hip fracture fixation with a sliding hip screw (SHS). The femoral head is primarily supplied by the medial femoral circumflex artery, and this circulation is typically preserved in intertrochanteric fractures, explaining why AVN is rarely observed in this setting. AVN is more often associated with trauma or systemic risk factors such as corticosteroid use or chronic alcohol abuse. We report a rare case of AVN in a previously healed intertrochanteric fracture treated with SHS, occurring after contralateral femoro-popliteal bypass surgery. A woman in her sixth decade presented with acute right hip pain one year after SHS fixation for an intertrochanteric femoral fracture. She had recently undergone contralateral femoro-popliteal bypass. Radiographs demonstrated femoral head collapse with screw penetration into the acetabulum, confirmed by CT. The patient underwent SHS removal and hybrid total hip arthroplasty. Histopathology revealed extensive coagulative necrosis of bone marrow with saponification and fibrosis, consistent with AVN. This case highlights a potential association between major vascular surgery and contralateral femoral head AVN after SHS fixation. Clinicians should maintain a high index of suspicion when new-onset hip pain develops following vascular procedures, as early recognition may offer an opportunity for joint-preserving management before irreversible collapse occurs.

## Introduction

Avascular necrosis (AVN) of the femoral head is a form of aseptic osteonecrosis resulting from the interruption of the blood supply to the proximal femur head, which is primarily derived from the medial femoral circumflex artery [1]. This terminal circulation makes the femoral head highly vulnerable to ischaemic injury, ultimately leading to osteocyte death and collapse. AVN is most commonly associated with traumatic events or atraumatic risk factors, such as corticosteroid use and chronic alcohol abuse.

In contrast, extracapsular fractures such as intertrochanteric fractures are generally managed with sliding hip screw (SHS) fixation and rarely result in AVN, since the femoral head circulation is typically preserved. Indeed, the reported incidence of femoral head AVN after intertrochanteric fracture fixation at two years is estimated at only 0.95-1.37% [2,3].

AVN may also arise in less typical scenarios, including after local intra-articular corticosteroid injection [4], in the setting of vascular malformations [5], or in association with subtle systemic vascular disorders such as sickle cell trait [6]. These examples highlight that the pathogenesis of AVN is multifactorial and that vascular compromise, whether direct, systemic, or remote, remains central to its development.

In this case report, we describe an unusual presentation of femoral head AVN occurring after SHS fixation in the context of a recent contralateral femoro-popliteal bypass surgery, a clinical scenario seldom reported in the literature. Given the reliance of femoral head vascularity on collateral pelvic circulation, contralateral vascular reconstruction may disrupt these pathways and inadvertently compromise ipsilateral hip perfusion.

## Case presentation

**Figure 1:**
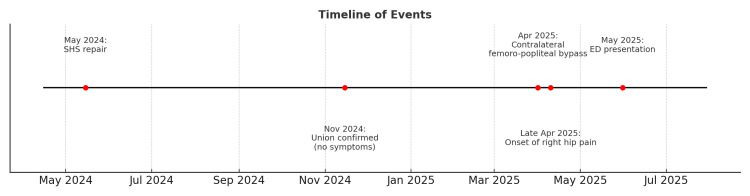
Timeline of Events SHS: sliding hip screw; ED: emergency department

A 65-year-old female presented to the Accident and Emergency Department with a three-week history of right hip pain. The pain had an acute onset and progressively worsened in the days leading up to her presentation. Twelve months earlier, she had sustained an ipsilateral intertrochanteric hip fracture managed with SHS fixation, with follow-up radiographs confirming union and an uneventful recovery. Six weeks prior to the presentation, she had undergone a contralateral femoro-popliteal bypass. There were no documented episodes of intra-operative or post-operative hypotension following the vascular procedure. Hip pain began approximately three weeks after the bypass procedure and continued to progress until her acute admission. She denied any further trauma in the interim (Figure 1).

**Figure 2:**
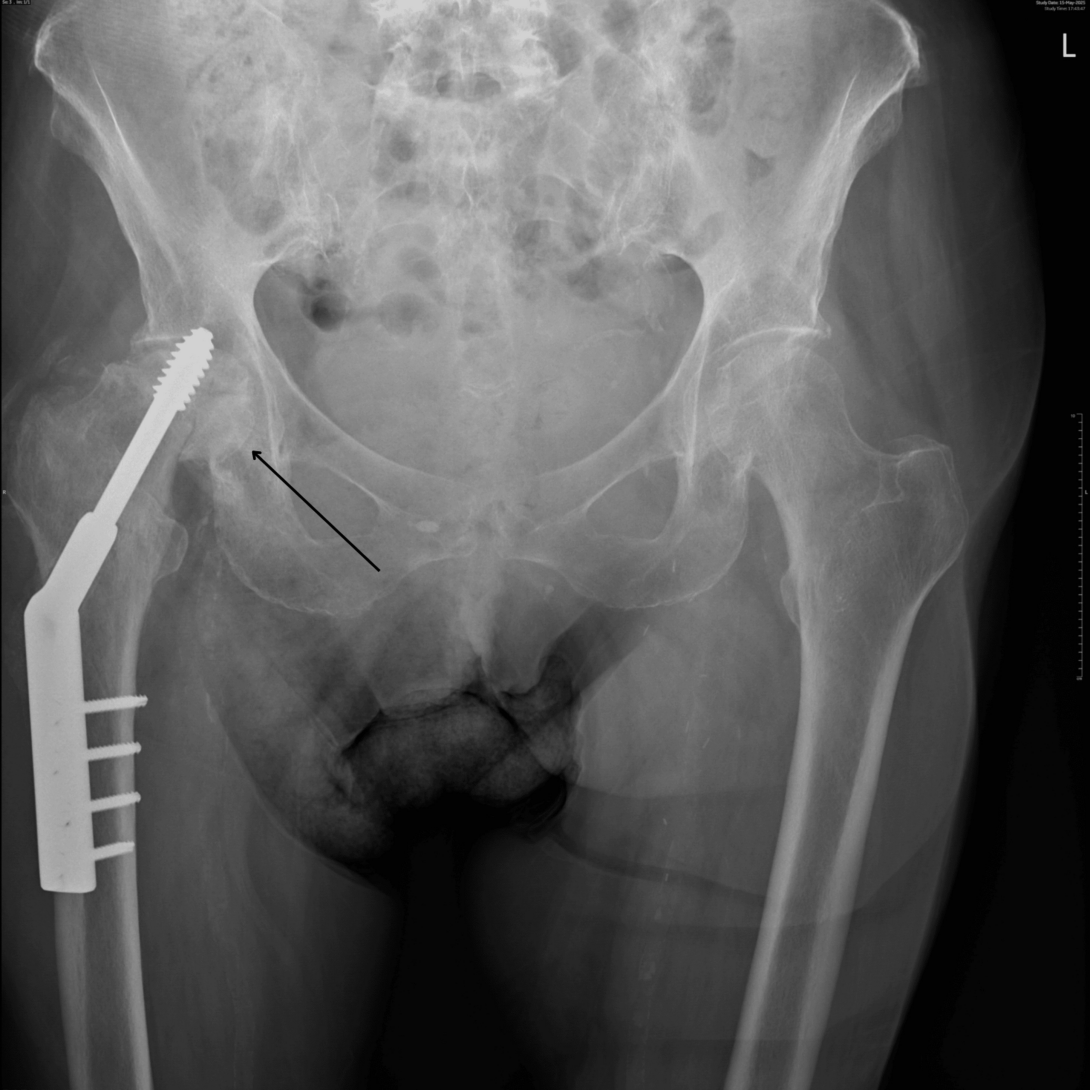
Pelvic X-ray Flattening of the femoral head with protrusion of the locking screw into the acetabulum.

**Figure 3:**
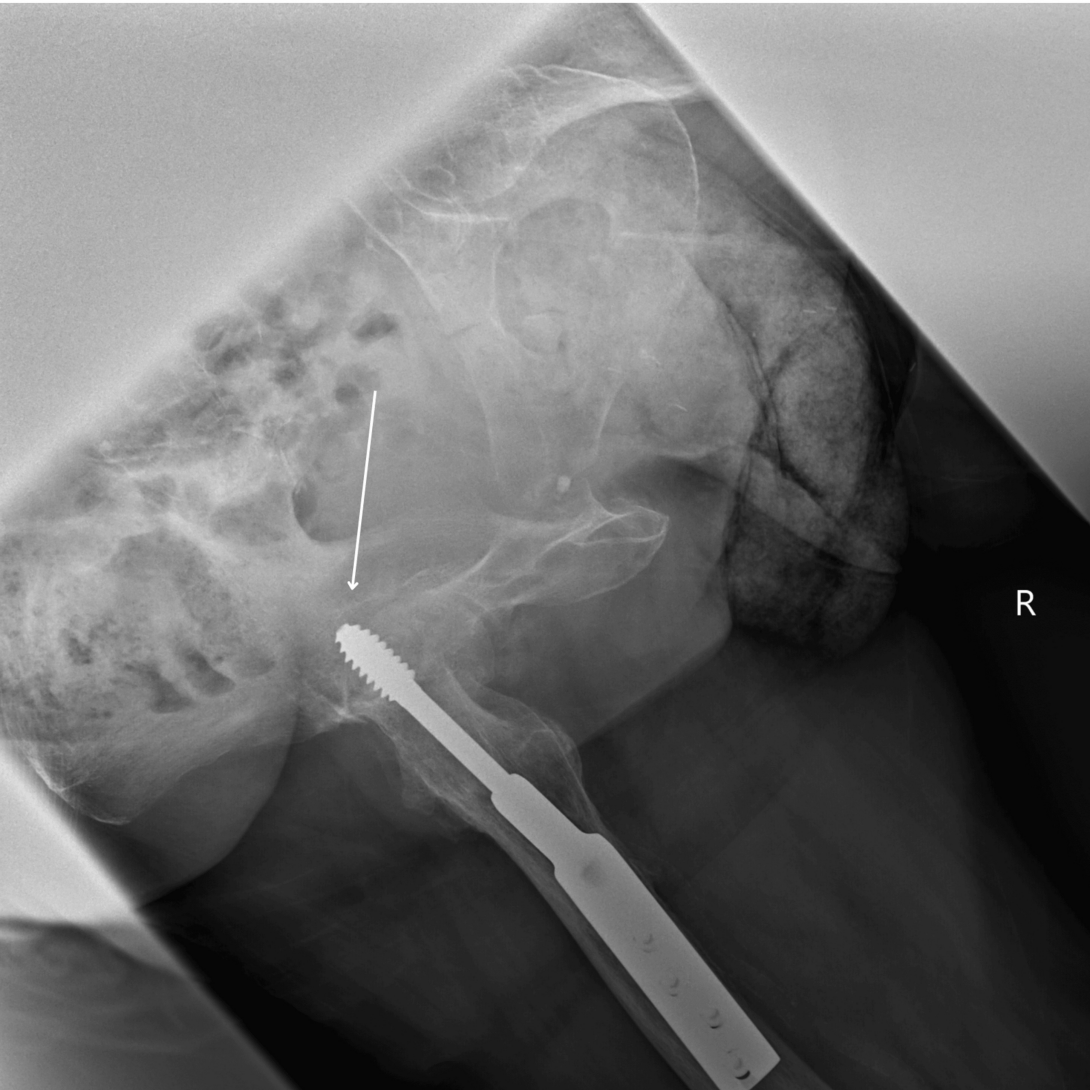
Right Lateral Hip X-ray Flattening of the femoral head with protrusion of the locking screw into the acetabulum.

A plain radiograph demonstrated flattening of the femoral head with protrusion of the locking screw into the acetabulum (Figures 2, 3).

**Figure 4:**
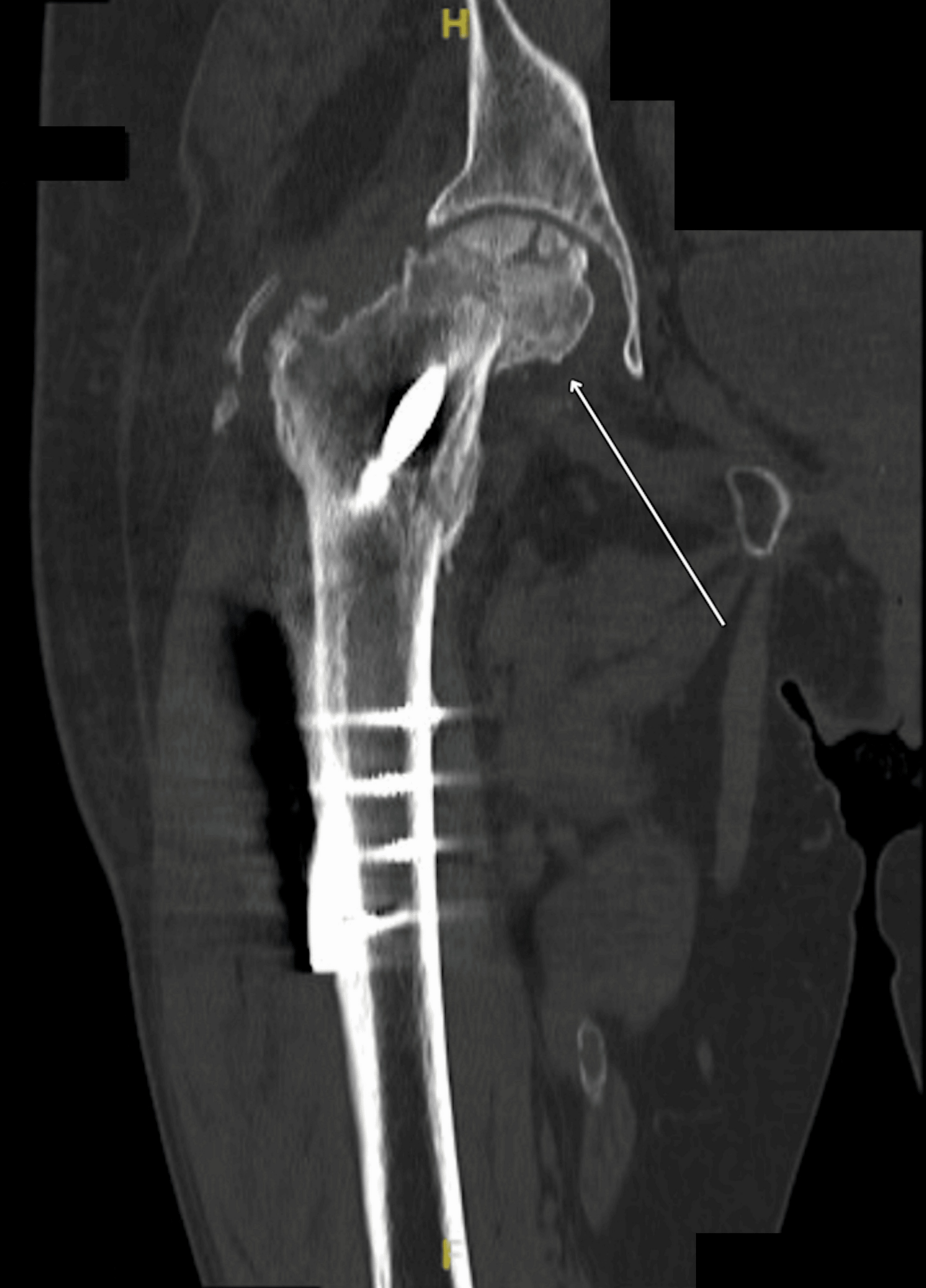
CT Right Hip Intracapsular neck of femur fracture associated with femoral head collapse, confirming hip AVN. AVN: avascular necrosis

A subsequent CT scan confirmed an intracapsular neck of femur fracture associated with femoral head collapse, confirming hip AVN (Figure 4).

**Figure 5:**
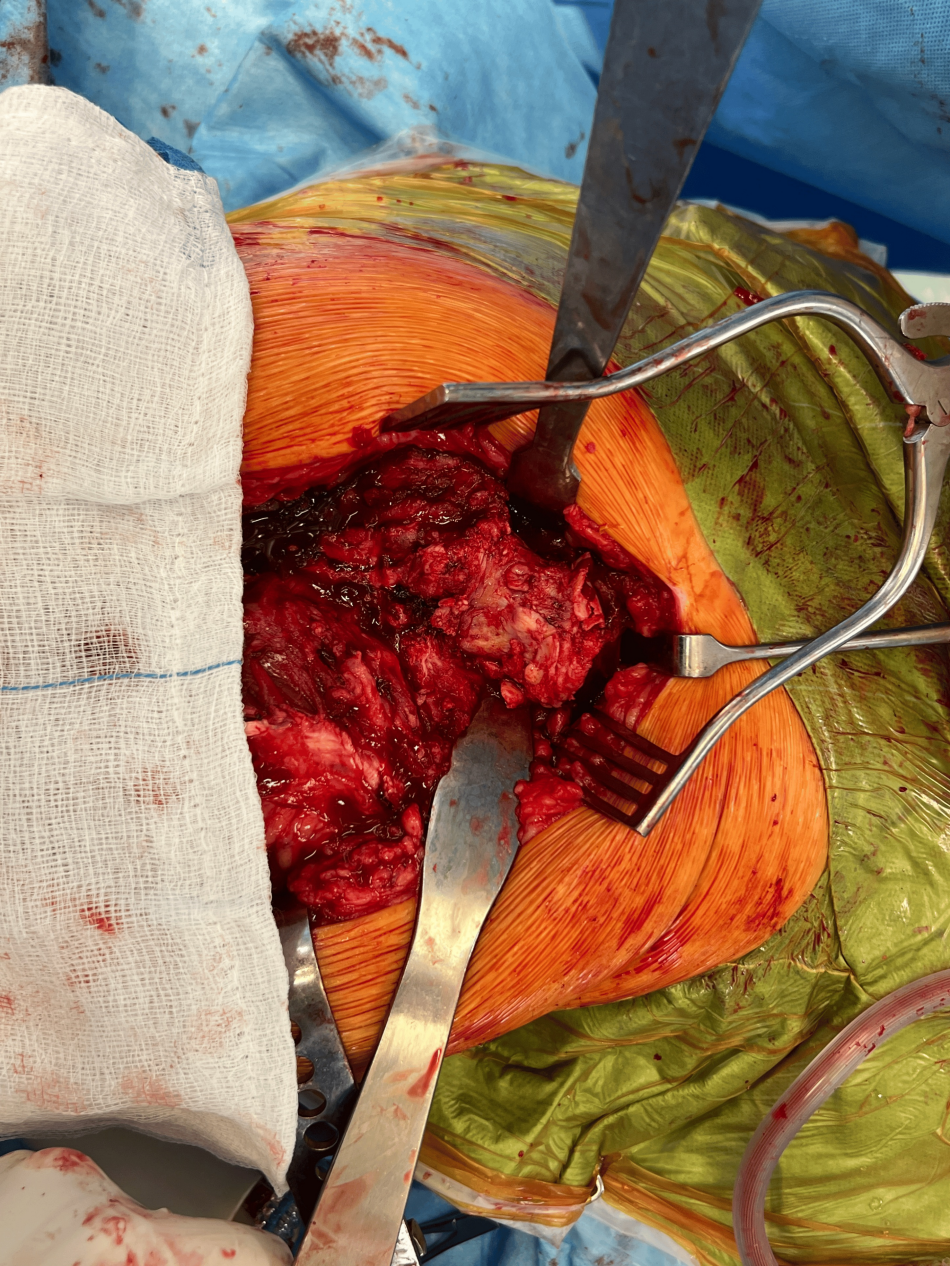
Intra-operative Right Hip Intra-operative image of the right hip demonstrating femoral head collapse with necrotic changes following previous sliding hip screw (SHS) fixation.

**Figure 6:**
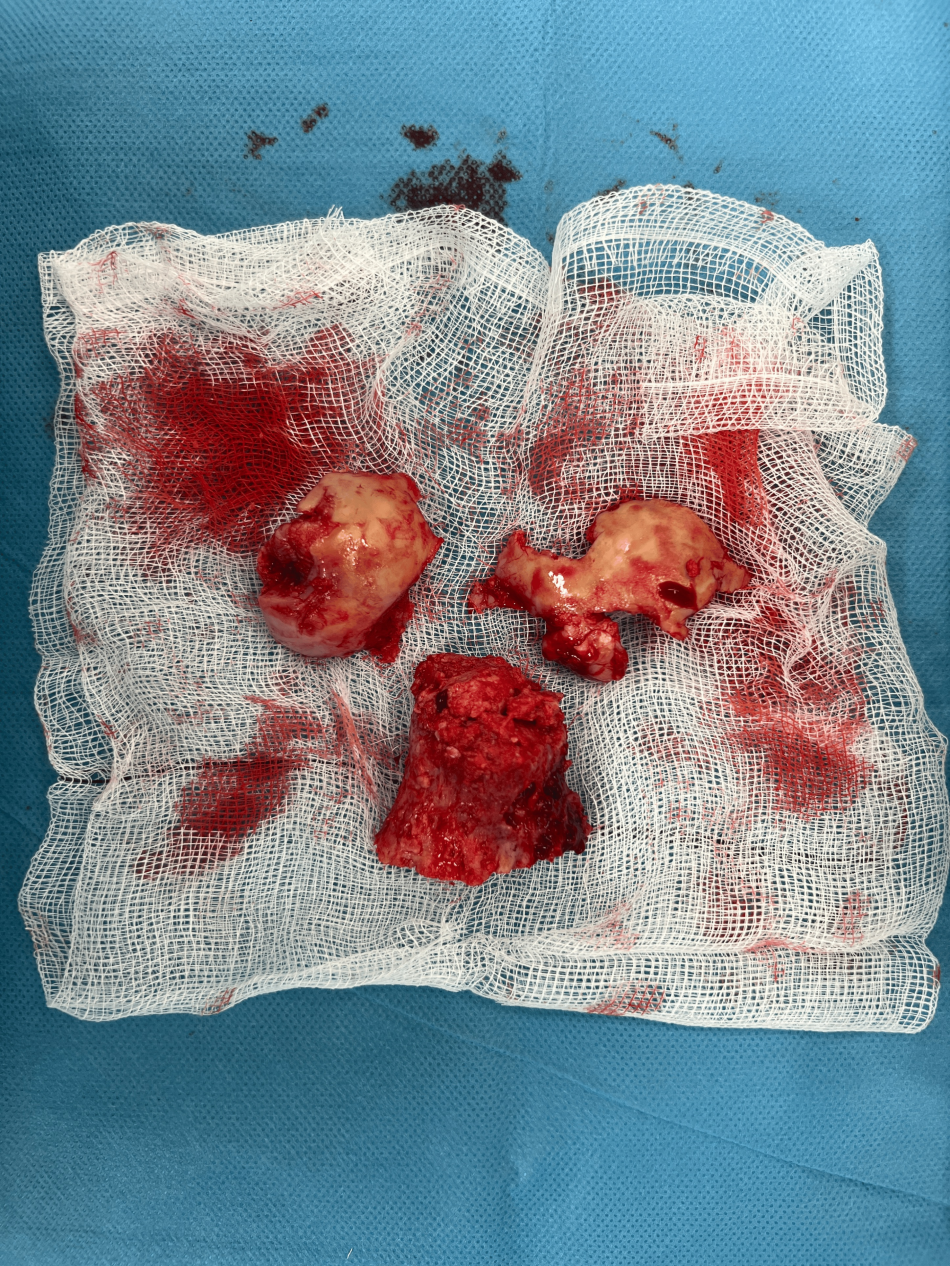
Right Femoral Head and Neck Intra-operative right hip image showing areas of avascular necrosis and loss of femoral head integrity.

The patient was admitted for further investigations and definitive management. Based on the patient's age and mobility, a decision was made to remove all current metalwork and perform a hybrid total hip arthroplasty (Figures 5, 6).

All excised tissue was sent for histopathological analysis. The report stated that there was 'extensive coagulative necrosis of marrow elements predominantly deep to the articular surface but also extending more deeply into the bone, with saponification of non-vital fatty bone marrow. Partly destroyed non-vital bone trabeculae present in the necrotic areas. Fibrosis is observed at the periphery of the areas of necrosis. There was no evidence of malignancy.'

**Figure 7:**
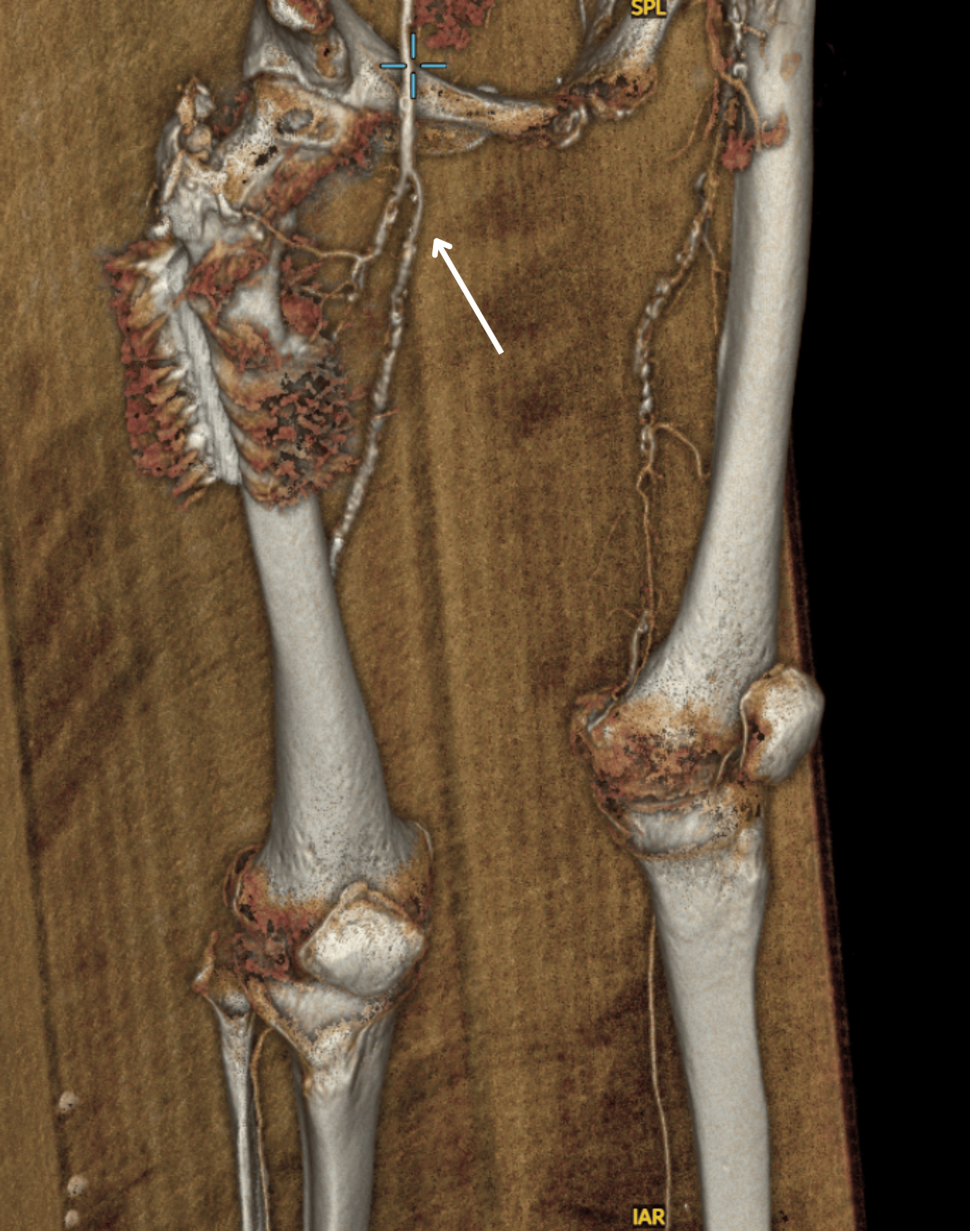
CT Angiography Normal patency of the abdominal aorta and iliac arteries. Mild non-stenotic mural calcification is observed in the wall of the infrarenal abdominal aorta and in the common iliac arteries; however, there is no significant aortoiliac stenosis. Left lower limb: The common femoral artery is patent and exhibits non-stenotic posterior wall calcification. The superficial femoral artery (SFA) occludes at its origin. The distal SFA and the suprageniculate popliteal artery reconstitute briefly for short segments. The proximal part of the popliteal artery re-occludes and reconstitutes more or less at the level of the distal femoral metaphysis, immediately proximal to the femoral condyles. The popliteal artery remains patent all the way to its bifurcation into the tibioperoneal trunk and the anterior tibial artery. Below the knee, the posterior tibial artery is patent all the way to the foot. The anterior tibial artery is also patent all the way to the foot. The peroneal artery occludes at the calf level. Right lower limb: The common femoral artery is patent and manifests a non-stenotic posterior wall calcified plaque. The superficial femoral artery is patent throughout its length with multifocal mural calcification but no significant stenosis or occlusion. The popliteal artery is also patent. Below the knee, there are three patent runoff vessels. Conclusion: Long left-sided femoro-popliteal artery occlusion.

The SHS had been inserted in February 2024 for a displaced intertrochanteric fracture of the right femur. One year later, the patient underwent a left common femoral artery endarterectomy and a femoro-popliteal bypass using the ipsilateral long saphenous vein on an elective basis. CT angiography of the lower limbs performed prior showed a congruent right hip joint with no evidence of AVN (Figure 7).

The patient was not on corticosteroids, bisphosphonates, or other medications known to predispose to or protect against AVN. No formal assessment of bone mineral density was performed; however, the initial hip fracture occurred following low-energy trauma, suggesting underlying fragility. Apart from this, there was no radiographic evidence of generalised osteopenia.

## Discussion

SHS fixation is a well-established method for treating intertrochanteric femoral fractures. These extracapsular fractures typically preserve the vascular supply to the femoral head. According to a systematic review by Barquet et al. (2014) [3], the incidence of AVN following SHS fixation is approximately 1%, underscoring the rarity of this complication. Therefore, when AVN develops in a patient with prior SHS fixation, it is not usually attributed to the surgical procedure alone.

In this case, the patient developed femoral head AVN, previously treated with SHS, shortly after undergoing a contralateral femoro-popliteal bypass. Although uncommon, similar cases have been reported. Shimatani et al. (2014) [6] described AVN following femoral artery stenting, attributing the event to compromised perfusion via the profunda femoris and medial femoral circumflex arteries, the key vessels supplying the femoral head. This illustrates how a direct vascular insult, even in the setting of previously intact circulation, may precipitate osteonecrosis.

Lykissas et al. (2012) [7] further suggested that major vascular surgery can provoke a systemic inflammatory and pro-thrombotic response, impairing microvascular flow and contributing to femoral head ischaemia, even contralaterally. Mallina et al. (2013) [2] also highlighted that vascular compromise, combined with mechanical stress, particularly in osteoporotic or previously weakened bone, may accelerate femoral head collapse. In our case, both impaired perfusion and increased mechanical loading likely acted synergistically in the pathogenesis of AVN.

Metabolic comorbidities such as diabetes mellitus and hypertension may further predispose patients to vascular vulnerability and risk of AVN. Diabetes is associated with microangiopathy, endothelial dysfunction, and impaired fibrinolysis, all of which can compromise femoral head perfusion [1]. Similarly, hypertension can accelerate atherosclerosis and reduce vascular compliance, limiting the collateral capacity of pelvic vessels [2]. Together, these systemic conditions may increase susceptibility to ischaemic events in the femoral head, particularly when compounded by major vascular interventions.

It is noteworthy that there was no clinical or radiological evidence of ipsilateral peripheral vascular disease, and the patient's prior fixation had demonstrated uneventful union. Furthermore, no ipsilateral femoral artery access was used during the angiogram or the vascular procedure, reducing the likelihood of a direct local vascular insult. This supports the hypothesis that the contralateral vascular intervention may have played a contributory role in the development of AVN.

Diagnosis of AVN can be delayed, as early clinical features are nonspecific and plain radiographs may be normal in the initial stages [8]. MRI remains the gold standard for early detection, capable of revealing bone marrow oedema and subchondral changes before structural collapse occurs [9]. As with many cases reported in the literature, the diagnosis was made at an advanced stage, by which time hip-preserving interventions were no longer viable, and total hip arthroplasty was required. While joint-preserving procedures such as femoral head revascularisation have been described [10], these are generally reserved for patients diagnosed before structural collapse. Roberts et al. (2025) [5] similarly demonstrated how advanced AVN progresses to fragmentation requiring arthroplasty, mirroring the outcome in our case.

In earlier stages of AVN, several joint-preserving options may be considered. These include core decompression, with or without biologic augmentation (such as bone marrow aspirate concentrate or stem cell therapy), which aims to reduce intraosseous pressure and stimulate revascularisation. Vascularised or non-vascularised bone grafting can also be employed in selected patients to restore mechanical support and promote healing. Pharmacological therapies such as bisphosphonates have shown variable results in delaying femoral head collapse, while anticoagulants may be beneficial in patients with underlying coagulopathy. These interventions are most effective when AVN is diagnosed before structural collapse, underscoring the importance of early detection and timely referral.

A limitation of this case is that no intraoperative tissue or metalwork samples were submitted for microscopy and culture. While the clinical and radiological features were not suggestive of infection, the absence of microbiological analysis means an infective cause cannot be definitively excluded.

This case underscores the importance of considering systemic vascular events as potential contributors to AVN, particularly in patients with recent major vascular interventions, even when the orthopaedic site is contralateral. It also highlights the need for multidisciplinary awareness and early imaging when symptoms arise. Prompt recognition may offer an opportunity for early intervention before irreversible joint damage occurs.

## Conclusions

This case underscores the importance of considering systemic vascular events as potential contributors to AVN, particularly in patients with recent major vascular interventions, even when the orthopaedic site is contralateral. While a temporal association was observed between contralateral femoro-popliteal bypass and subsequent AVN in our patient, causality cannot be definitively established. Other factors, including diabetes, hypertension, underlying microvascular compromise, and implant-related mechanical stress, may also have played a role.

Although direct reports of contralateral hip AVN following vascular procedures are rare, emerging evidence supports a plausible pathophysiological link. The femoral head's blood supply, primarily from the medial femoral circumflex artery, is highly vulnerable to disruption. Vascular interventions, even when performed on the opposite limb, may trigger systemic or local haemodynamic changes that compromise femoral head perfusion. This highlights the importance of assessing vascular status in both lower limbs before and after major vascular surgery. Compromise of the medial femoral circumflex system can result in osteonecrosis irrespective of the side of intervention. Early recognition and timely orthopaedic management, often surgical, are essential to relieve pain and restore function. Heightened clinical vigilance and interdisciplinary awareness are critical, as the overlap between vascular and orthopaedic pathology, though uncommon, can have significant functional consequences if not promptly addressed.
